# Impaired awareness of hypoglycemia in children and adolescents with type 1 diabetes mellitus in north of Jordan

**DOI:** 10.1186/s12902-019-0441-9

**Published:** 2019-10-24

**Authors:** Mohammad J. Alkhatatbeh, Nedaa A. Abdalqader, Mohammad A. Y. Alqudah

**Affiliations:** Department of Clinical Pharmacy, Faculty of Pharmacy, Jordan University of Science and Technology, Irbid, 22110 Jordan

**Keywords:** Awareness, Children, Hypoglycemia, Type 1 diabetes

## Abstract

**Background:**

Hypoglycemia is a common complication of insulin therapy in patients with Type 1 Diabetes Mellitus (DM). Awareness of hypoglycemic symptoms helps patients to recognize hypoglycemia and initiate self-treatment. Impaired Awareness of Hypoglycemia (IAH) exposes patients to severe hypoglycemia, which could be associated with seizures and unconsciousness. This study aimed to assess IAH, frequency of hypoglycemia, severe hypoglycemia and intensity of hypoglycemic symptoms among children and adolescents with Type 1 DM in North of Jordan.

**Methods:**

Data were collected from 94 children and adolescents with Type 1 DM. Clarke’s and Edinburgh surveys were used to assess IAH and individual symptoms of hypoglycemia, respectively. Frequency of hypoglycemia and other related information were obtained by self-reporting or from medical records.

**Results:**

16.0% of participants were having IAH, 66.0% of participants reported recurrent hypoglycemia (>once/month) and 18.0% of participants developed ≥1 severe hypoglycemia during the previous year. IAH was not associated with age, gender, duration of DM, HbA1c, insulin regimen, adherence to insulin or development of severe hypoglycemia (*p*-values> 0.05). Instead, IAH was associated with frequency of hypoglycemia during the previous 6 months (*p*-value< 0.01). Hunger, tiredness, dizziness, drowsiness, inability to concentrate, trembling and weakness were the most common symptoms felt by participants when they develop hypoglycemia. Hunger was the only common symptom that was significantly higher in children compared to adolescent (*p*-value < 0.01).

**Conclusions:**

This study has reported low prevalence of IAH in children and adolescents with Type 1 DM in North of Jordan. IAH was more common in subjects with more frequent hypoglycemia.

## Background

Type 1 Diabetes Mellitus (DM) is usually diagnosed in children and adolescents and is characterized by pancreatic beta-cell dysfunction and impaired insulin secretion [1, 2]. Hypoglycemia, which is defined as a blood glucose concentration of less than 70 mg/dL [3], is considered as a common acute complication of insulin therapy [4]. Children and adolescents with Type 1 DM may develop hypoglycemia because of increased glucose utilization that results from excessive insulin dosing, skipping meals or reduced insulin requirements during exercise [4, 5]. Fortunately, hypoglycemia is accompanied by alarming symptoms that are caused by neuroglycopenia and stimulation of the autonomic nervous system, which is triggered by the counter regulatory hormone responses [6]. Feeling of these symptoms helps children, adolescents and their parents to recognize hypoglycemia and initiate self-treatment with oral carbohydrate intake [6]. This can restore blood glucose to normal levels and prevent further consequences of hypoglycemia [7].

Importantly, children and adolescents who experience frequent hypoglycemia may get defective counter-regulatory hormone responses that result in reduced feeling of hypoglycemia; a condition called Impaired Awareness of Hypoglycemia (IAH) [8]. Impairment of cognition that could also be associated with hypoglycemia may prevent children from asking for treatment [9]. So, inability to recognize hypoglycemia exposes children and adolescents to more severe hypoglycemia that could be associated with seizures and loss of consciousness [10, 11]. Consequently, children and adolescents with IAH may experience fear of developing hypoglycemia, which may affect their adherence to insulin regimens and hence the achievement of a good glycemic control [12]. This may result in persistent hyperglycemia and the development of long-term micro- and macro-vascular DM complications including nephropathy, neuropathy, retinopathy and atherosclerosis [13].

Prevalence of IAH in children and adolescents with Type 1 DM has not been studied previously in Jordan. In this study, our purpose was to assess IAH in children and adolescents with Type 1 DM recruited from outpatient pediatric clinics of the major specialized pediatric public hospital in North of Jordan. We aimed to assess the intensity of hypoglycemic symptoms, to determine the prevalence of IAH and to find associations between IAH and other variables including age, gender, body mass index (BMI), hemoglobin A1c (HbA1c), duration of DM, insulin regimens, glycemic control, frequency of hypoglycemia and history of developing severe hypoglycemia.

## Methods

This is a cross-sectional study that was conducted at outpatient clinics of Princess Rahma Pediatric Hospital (PRPH) in Irbid, Jordan. This public hospital is the only pediatric referral hospital in North of Jordan that receives cases from four governorates (Irbid, Ajloun, Jerash and Mafraq). Ethical approval was obtained from the Institutional Research Board (IRB) of the Ministry of Health before starting patients’ recruitment (Reference code: MOH REC160136).

### Participants

Out of 130 children and adolescents (≤ 16 years old) with Type 1 DM who regularly receive treatment in outpatient pediatric clinics of PRPH, 94 patients from both genders, who were on insulin therapy for a minimum of 6 months, were included in the current study. The rest of patients (*n* = 36) were excluded from the study because their duration of insulin therapy was less than 6 month or because they were not mature enough to express their feeling of hypoglycemia (age < 5 years). So, the age of our study participants ranged from 5 to 16 years. Sample size was calculated based on the prevalence of IAH in children and adolescents with Type 1 DM that was determined previously by Graveling AJ et al. using Clarke’s hypoglycemia survey [9]. The following formula was used for this purpose: *n* = [(z)^2^ (p) (1-p)/(d)^2^]/(1 + [(z)^2^ (p) (1-p)/(d)^2^]/N) [14] where n is the sample size, t is 1.96 (z-value corresponding to 95% confidence interval), p is 0.22 (the estimated prevalence of IAH as determined by Graveling AJ et al. [9], d is 0.05 (the acceptable margin of error based on a confidence interval of 95 and 5% error) and N is 130 (the population size). Consequently, the calculated sample size was 87 but we tried to recruit larger number of participants (94) which was comparable to the sample size of Graveling AJ et al. [9]. Parents of participants who agreed to participate in the study had signed the assent forms. Well-validated questionnaires to assess hypoglycemia awareness and intensity of hypoglycemic symptoms were applied to participants and/or their parents. A well-trained researcher was available to explain the purpose of the study and answer any inquiry revealed by participants and/or their parents. Participants were divided according to their age into children (< 10 years) and adolescents (≥10 years) according to the World Health Organization’s definition of adolescence [15].

### Data collection

Data were collected between September 2016 and March 2017. Age, gender, duration of Type 1 DM, duration of insulin therapy and HbA1c levels were obtained from medical records. Information about adherence to insulin regimen, maintenance of regular meals after insulin injection, blood glucose monitoring, frequency of blood glucose measurements below 70 mg/dL during the previous 6 months and development of severe hypoglycemia that required assistance from others or was associated with seizures or unconsciousness during the previous year were obtained by self-reporting of participants and their parents. BMI was calculated using the formula: BMI = body weight (Kg)/height (m^2^).

### Determining level of hypoglycemic awareness

Level of hypoglycemic awareness was determined using Clarke’s hypoglycemia survey [9, 16]. The survey was translated from English to Arabic using the standard forward-backward translation method by two independent translators. The last version of the survey was assessed for face and content validity by a group of experts in the field of DM and questionnaire design before pilot testing. The survey involved eight questions that evaluated patient’s ability to recognize hypoglycemia. Response to each question was determined as either aware (A) or reduced aware (R). Patients who reported ≥4 R responses were classified as having IAH while patients who reported ≤3 R responses were classified as hypoglycemic aware [8, 9].

### Intensity of hypoglycemic symptoms

Edinburgh hypoglycemia survey [17] was used to assess the intensity of individual hypoglycemic symptoms. The survey was translated from English to Arabic and pilot tested using the same method mentioned in the previous section. The survey is based on scoring the usual feeling of each symptom using a scale from 1 to 7 (1 represents minimal feeling while 7 represents maximum feeling). The survey included the following symptoms: anxiety, confusion, blurred vision, dizziness, difficulty in speaking, drowsiness, hunger, headache, inability to concentrate, nausea, sweating, pounding heart, tiredness, tingling lips, trembling, warmth and weakness.

### Statistical analysis

IBM SPSS statistics software version 20 (Armonk, New York, USA) was used to perform statistical analysis. Categorical data were expressed as participants’ number (percentage). Continuous variables, that were not normally distributed, were expressed as median (interquartile range). Mann Whitney U test was used to determine differences in continuous variables between children and adolescents. Chi-square test or Fisher’s exact test was used to determine association between categorical variables as appropriate. All *p*-values were two-tailed and considered statistically significant at < 0.05.

## Results

### General characteristics

Data were collected from 94 children and adolescents with Type 1 DM from both genders (51 males and 43 females). The median (interquartile range) of age was 10 (8–13) years and the median (interquartile range) duration of Type 1 DM and insulin therapy was 2 (1–4) years. General characteristics for both children and adolescents are shown in Table 1.

**Table 1:** General Characteristics

Variable	Children and adolescents (5–16 years old) (*n* = 94)	Children (5–10 years old) (*n* = 52)	Adolescents (10–16 years old) (*n* = 42)	*P*-value^*^
Age (Years)	10 (8–13)	8 (6–10)	13 (12–14)	–
Gender (male/female)	51/43	29/23	22/20	–
BMI (Kg/m^2^)	19.4 (16.6–23.4)	17.6 (15.4–20.4)	22 (18.3–24.3)	< 0.01
Duration of DM or insulin therapy (Years)	2 (1–4)	1.8 (1–3)	3 (1–6)	< 0.01
HbA1c (%)	8.4 (7.5–9.1)	8 (7–9)	9 (8–10)	0.01
Insulin regimen				
Mixtard insulin (regular insulin + isophane insulin)	88 (93.6)	50 (96.2)	38 (90.5)	0.40
Regular insulin + Insulin Glargine	6 (6.4)	2 (3.8)	4 (9.5)	
Adherence to insulin administration				
Not completely adherent	29 (30.9)	15 (28.8)	14 (33.3)	0.64
Completely adherent	65 (69.1)	37 (71.2)	28 (66.7)	
Maintaining regular meals after insulin administration				
Always	69 (73.4)	44 (84.6)	25 (59.5)	0.01
Sometimes	25 (26.6)	8 (15.4)	17 (40.5)	
Frequency of hypoglycemia during the previous 6 months				
≤ Once /month	32 (34)	17 (32.7)	15 (35.7)	0.83
> Once/month	62 (66)	35 (67.3)	27 (64.3)	
Development of severe hypoglycemia during the last year				
Yes	17 (18.1)	11 (21.2)	6 (14.3)	0.43
No	77 (81.9)	41 (78.8)	36 (85.7)	

^*^ Mann Whitney U test was used to find differences between children and adolescents in continuous variables, Chi-square test or Fisher’s exact test were used to find association between age groups and categorical variables (*P* < 0.05 is considered significant). Data are expressed as median (25th–75th percentile) for continuous variables and as frequency (%) for categorical variables. DM; Diabetes Mellitus. Data are expressed as median (interquartile range) or as frequency (%)

### Frequency of hypoglycemia

All participants had reported that they self-monitor their blood glucose level at home on a daily basis. Recurrent hypoglycemia (reporting blood glucose measurements below 70 mg/dL more than once per month in the previous 6 months) was reported by 66.0% of participants. 18.1% of participants had also reported that they developed ≥1 severe hypoglycemic episode that required assistance from others or was associated with seizures or unconsciousness during the previous year.

### Prevalence of impaired awareness of hypoglycemia

The prevalence of IAH among children and adolescents with Type 1 DM was 16.0%. As shown in Table 2, level of hypoglycemia awareness was not significantly associated with patient’s age, gender, BMI, duration of Type 1 DM, duration of insulin therapy, HbA1c, insulin regimen, adherence to insulin administration, maintaining regular meals after insulin administration or development of severe hypoglycemia during the previous year (*p*-values > 0.05). Instead, the level of hypoglycemic awareness was only significantly associated with the frequency of hypoglycemia during the previous 6 months (*p*-value < 0.01).

**Table 2:** Association of hypoglycaemia awareness determined by Clarke’s method with different variables

	Aware (*n* = 79)	IAH (*n* = 15)	*P*-value^*^
Age (Years)	10 (8–13)	10 (5–11)	0.13
Gender			
Male	42 (53.2)	9 (60)	0.78
Female	37 (46.8)	6 (40)	
BMI (Kg/m^2^)	19.5 (16.6–23.9)	17.8 (16.5–23)	0.36
Duration of DM (Years)	2 (1–4)	1 (1–7)	0.48
Duration of insulin therapy (Years)	2 (1–4)	1 (1–7)	0.48
HbA1c (%)	8.5 (7.7–9.2)	8 (6–9)	0.12
Insulin regimen			0.56
Mixtard insulin (regular insulin + isophane insulin)	73 (92.4)	15 (100)	
Regular insulin + Insulin Glargine	6 (7.6)	0 (0)	
Adherence to insulin administration			
Not completely adherent	28 (35.4)	1 (6.7)	
Completely adherent	51 (64.6)	14 (93.3)	0.06
Maintaining regular meals after insulin administration			
Always	58 (73.4)	11 (73.3)	1.00
Sometimes	21 (26.6)	4 (26.7)	
Frequency of hypoglycemia during the previous 6 months			
≤ Once/month	32 (40.5)	0 (0)	< 0.01
> Once/month	47 (59.5)	15 (100)	
Development of severe hypoglycemia during the previous year			
Yes	12 (15.2)	5 (33.3)	0.14
No	67 (84.8)	10 (66.7)	

^*^ Mann Whitney U test was used to find differences between aware and IAH participants in continuous variables, Chi-square test or Fisher’s exact test were used to find association between hypoglycemia awareness and categorical variables (*P* < 0.05 is considered significant). Data are expressed as median (25th–75th percentile) for continuous variables and as frequency (%) for categorical variables. IAH: impaired awareness of hypoglycemia

### Intensity of hypoglycemic symptoms

As shown in Table 3, hunger and tiredness were the most intense hypoglycemic symptoms that were usually felt by participants (median intensity score was 7 for hunger and 6 for tiredness). Dizziness, drowsiness, inability to concentrate, trembling, weakness and confusion were moderately felt by participants when they develop hypoglycemia (median intensity score was 5 for all of these symptoms and 4 for confusion). Other hypoglycemic symptoms including anxiety, blurred vision, difficulty in speaking, headache, nausea, pounding heart, sweating, tingling lips and warmth were the least intense hypoglycemic symptoms that were felt by participants (median intensity score was ≤3). Feeling hunger, the most common hypoglycemic symptom, was significantly higher among children compared to adolescents (*p*-value < 0.01).

**Table 3:** Intensity of hypoglycaemic symptoms determined by Edinburgh survey in children and adolescents with Type 1 Diabetes Mellitus

Hypoglycemic symptoms	Intensity of symptom^*^	Age Category			
		Intensity of symptoms ^*^ in Children (5–10 years old) (*n* = 52)	Intensity of symptoms ^*^ in Adolescents (10–16 years old) (*n* = 42)	Mann-Whitney U	*P*-value^¥^
Anxiety	1 (1–2)	1 (1–1)	1 (1–3)	892.5	0.07
Blurred vision	1 (1–4)	1 (1–2)	1.5 (1–5)	834.5	0.02
Confusion	4 (1–5.3)	3 (1–5.75)	4 (1–5.25)	994.5	0.45
Difficulty in speaking	1 (1–2)	1 (1–2)	1 (1–1)	882.5	0.05
Dizziness	5 (1–7)	4 (1–6)	6 (2.75–7)	871	0.08
Drowsiness	5 (4–7)	5 (2–7)	5 (4–7)	988.5	0.42
Headache	3 (1–6)	3 (1–6)	2 (1–5)	1042.5	0.70
Hunger	7 (5–7)	7 (6–7)	5 (2–7)	638.5	< 0.01
Inability to concentrate	5 (2.8–7)	4 (1–6)	5 (4–7)	869	0.08
Nausea	1 (1–1)	1 (1–1)	1 (1–1)	1078.5	0.89
Pounding heart	1 (1–5)	1.5 (1–5)	1 (1–2.75)	919	0.14
Sweating	2 (1–5)	2 (1–5)	3 (1–5.5)	952.5	0.27
Tingling lips	1 (1–3)	1 (1–3.75)	1 (1–1.5)	1078	0.89
Tiredness	6 (4–7)	6 (4–7)	5 (4–7)	893.5	0.12
Trembling	5 (1–7)	6 (1–7)	5 (1–7)	1012.5	0.53
Warmth	1 (1–6)	1 (1–4)	2 (1–6)	843.5	0.04
Weakness	5 (3–7)	5 (2–7)	4 (3.75–6)	1006.5	0.51

^*^ Median (25th – 75th percentiles), ^**¥**^ Mann Whitney U test (*P* < 0.05 is considered significant). Data for continuous variables are expressed as median (25th – 75th percentiles)

## Discussion

The present study has reported low prevalence of IAH (16.0%) among children and adolescents with Type 1 DM treated in the main public pediatric hospital in North of Jordan using Clarke’s hypoglycemia survey [9, 16]. This was less than the prevalence determined by Graveling AJ et al. (22.4%) and Ly TT et al. (29%) studies [8, 9], which used the same hypoglycemia survey in children and adolescents with Type 1 DM. Additionally, Abraham MB et al. [18] reported reduced prevalence of IAH in children with Type 1 DM from 33% in 2002 to 21% in 2015, which was still higher than the prevalence reported in the current study. Even, the prevalence of IAH in our study was also lower than the prevalence of IAH reported in adults with Type 1 DM. For example, the prevalence of IAH was 28% as determined by Høi -Hansen T et al. [19].

Although there was no significant difference in the duration of Type 1 DM between hypoglycemia aware and IAH participants (Table 2), a possible explanation for the low prevalence of IAH in the current study compared to other studies [8, 9, 18] is the relatively shorter duration of Type 1 DM (The median (interquartile range) duration of Type 1 DM was only 2 (1–4) years). It has been reported that increased duration of DM is associated with increased risk of IAH [20]. Patients with longer duration of DM may have experienced more frequent hypoglycemia, which can blunt glucagon and adrenergic responses to hypoglycemia and thus increases patient’s risk to hypoglycemia [20]. So, patient who experience recurrent hypoglycemia are expected to be at increased risk of having IAH [20]. This was confirmed by the current study as IAH was significantly associated with the frequency of hypoglycemia during the previous 6 months. As shown in Table 2, all participants who were classified as having IAH reported that they had more frequent hypoglycemia compared to hypoglycemic aware patients. This finding was consistent with Ly TT et al. [8] study, in which more patients with recurrent hypoglycemia reported IAH. The justification is that recurrent hypoglycemia can reduce the blood glucose level that stimulates counter regulatory hormone responses required to restore normal blood glucose level in the following episode of hypoglycemia [20]. Thus, patients may develop hypoglycemia without stimulating counter regulatory hormone responses, which are responsible for the development of hypoglycemic symptoms [20]. Consequently, this could predispose patients to the development of severe hypoglycemia, which requires assistance from other persons to treat [10]. However, the current study did not show significant association between level of hypoglycemic awareness and the development of severe hypoglycemia during the previous year (Table 2). This finding was inconsistent with Ly TT et al. [8] study, which found that patients with IAH had experienced higher rate of severe hypoglycemia compared to hypoglycemic aware patients. As well, Abraham MB et al. [18] study had shown that IAH was associated with increased risk of developing severe hypoglycemia.

Another explanation for the low prevalence of IAH in the current study is the poor glycemic control reported for our study participants. As shown in Table 1, the median (interquartile range) of HbA1c was 8.4% (7.5–9.1%). As well, and 68.1% of the participants were having HbA1c levels > 7%, which indicates that around 2/3 of participants were having uncontrolled blood glucose levels. It is expected that patients with IAH should have lower HbA1c levels compared to aware patients because frequency of hypoglycemia is associated with lower glycemic control [20]. However, the current study did not show significant difference in HbA1c level between IAH and hypoglycemia aware participants. This was consistent with Graveling AJ et al. study [9] and inconsistent with Ly TT et al. study [8], in which patients with IAH were having significantly lower level of HbA1c compared to hypoglycemia aware patients. In general, the average HbA1c level in both studies [8, 9] was > 8%, suggesting that their participants were also having uncontrolled blood glucose levels.

The current study has also reported intensity of hypoglycemic symptoms in children and adolescents with Type 1 DM. As shown in the results, hunger and tiredness were the most intense symptoms while dizziness, drowsiness, inability to concentrate, trembling, weakness and confusion were of moderate intensity. Similarly, Amin et al. [21] had reported that hunger, trembling, weakness and tiredness were commonly felt by children with Type 1 DM when they develop hypoglycemia. There was no significant difference in the intensity score for each of these symptoms between children and adolescents except for hunger, which was significantly higher in children compared to adolescents (Table 3).

Collectively, the current study has determined the prevalence of IAH in children and adolescents with Type 1 DM in North of Jordan. The main findings that add to the literature were the low prevalence of IAH in children and adolescents with Type 1 DM compared to other similar studies [8, 9, 18] and the significant association between the development of IAH and the frequency of hypoglycemia during the past 6 months. Although data in this study were collected using well-validated surveys over a period of 6 months, this study has some limitations. First, children and adolescents with Type 1 DM were recruited from a single hospital in the country. However, this hospital is public and is considered as the major pediatric hospital in North of Jordan. Second, data in this study were collected by self-reporting which is the same method used in other similar studies [8, 9, 18]. Because hypoglycemia could be underappreciated [22], patients may underreport hypoglycemia and this could affect the true prevalence of hypoglycemia and IAH. Although the calculated sample size appears to be relevant, the small number of participants compared to other studies [8] may not be enough to report significant associations between IAH and other expected variables including HbA1c and severe hypoglycemia. As well, the exclusion of children who are < 5 years old may also be considered as a limitation when we compare our results to other studies that they did not consider this exclusion. Despite of these limitations, results of this study can still add new information about the prevalence of IAH in children and adolescents with Type 1 DM in North of Jordan. As well, results of this study may encourage future research about hypoglycemia in patients with Type 1 DM and how to enhance the level of hypoglycemic awareness in this population.

## Conclusions

The current study has reported low prevalence of IAH in children and adolescents with Type 1 DM in North of Jordan. There was a significant association between the development of IAH in children and adolescents and the frequency of hypoglycemia during the past 6 months; suggesting that IAH was more common in subjects with more frequent hypoglycemia. This study has also reported hunger and tiredness as the most intense symptoms in children and adolescents compared to other symptoms of hypoglycemia.

## Data Availability

Data and materials of this study are available from the corresponding author on reasonable request.

## Abbreviations

BMI: Body Mass Index
DM: Diabetes Mellitus
HbA1c: Hemoglobin A1c
IAH: Impaired Awareness of Hypoglycemia
IRB: Institutional Research Board
PRPH: Princess Rahma Pediatric Hospital
